# Plume influence analysis of small bipropellant thruster on solar array of GEO satellite

**DOI:** 10.1371/journal.pone.0199667

**Published:** 2018-09-04

**Authors:** Kyun Ho Lee

**Affiliations:** Department of Aerospace Engineering, Sejong University, Seoul, Republic of Korea; Virginia Commonwealth University, UNITED STATES

## Abstract

An exhaust plume gas flow from a bipropellant thruster has been recognized as a potential source of a load, heat, and contamination on the exposed satellite components which can especially degrade the optical properties and performance of solar panel. Therefore, accurate predictions of a bipropellant thruster plume gas flow and assessments of its influences should be considered at the design phase of a satellite. The objective of the present study is to investigate the plume gas flow behavior of a small bipropellant thruster and to evaluate its influence on the solar array of a GEO satellite numerically. To deal with complex plume flow regimes efficiently, the combined approach of computational fluid dynamics (CFD) and Direct Simulation Monte Carlo (DSMC) was applied depending on the flow characteristic conditions. Throughout the numerical results of the present study, the influences of the plume gas flow exhausted from a single bipropellant thruster were considered for the disturbance, heat, and contamination of the three-dimensional satellite configuration equipped with a large solar array.

## Introduction

**Fig 1:**
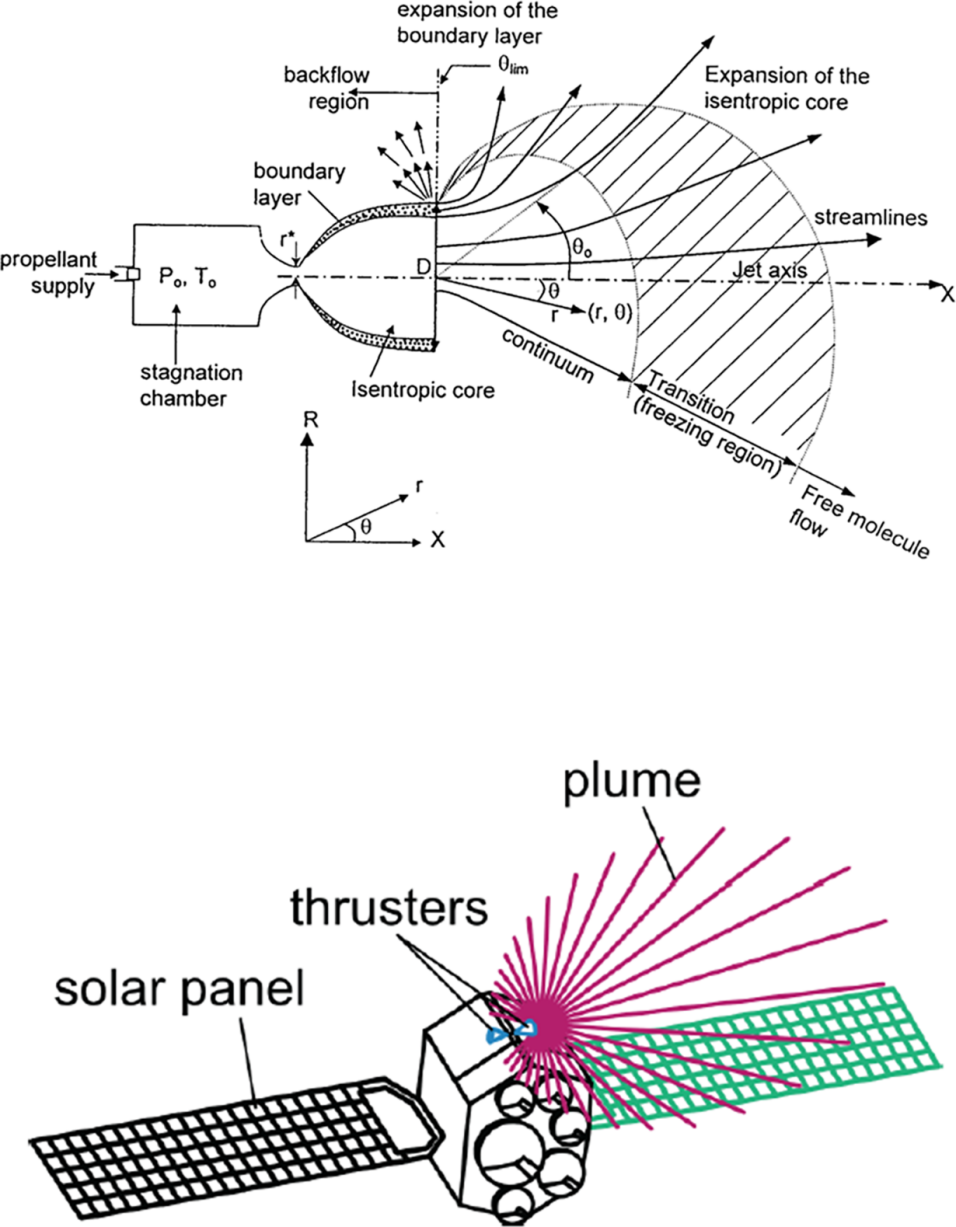
Satellite thruster plume flow behavior in a vacuum space [1]. (A) Complex plume flow regimes. (B) Thruster plume influence on the satellite.

A space propulsion system plays an important role for the attitude control, drag make-up and orbit transfer maneuvers of a satellite by ejecting a propellant gas at a very high speed into a vacuum space. A space propulsion system can be classified into several types based on the energy source for thrust production. Among them, a bipropellant thruster using a chemical reaction of a fuel and an oxidizer is frequently employed for the primary propulsion system of a geosynchronous (GEO) satellite especially to compensate for various perturbations and to maintain the nominal position of the GEO satellite within specified longitudinal and latitudinal bounds during a mission period. Generally, periodic firings of the bipropellant thruster produce a high-temperature and high-pressure plume gas flow which diffuses freely into a vacuum space along all directions shown in Fig 1 [1]. Thus, this exhaust plume gas flow from the bipropellant thruster has been recognized as a potential source of heat and contamination on the exposed satellite components which can especially degrade the optical properties and performance of solar panels. Therefore, accurate predictions of the thruster plume gas flow and assessments of its influences should be considered from the initial design step of a satellite [1,2].

Thus, the present research numerically investigated the plume gas flow behavior of a small bipropellant thruster and evaluated its influence on the solar array of a GEO satellite. To deal with the complex plume flow regimes of a stagnation, a continuum, and a rarefied condition efficiently, the combined approach of computational fluid dynamics (CFD) and Direct Simulation Monte Carlo (DSMC) was applied depending on the flow conditions. Throughout the numerical results of the present study, the influences of the plume gas flow exhausted from a single bipropellant thruster were considered for the disturbance, heat, and contamination of a three-dimensional satellite configuration equipped with a large solar array.

## Numerical methodology

### Navier-Stokes equations

To calculate the continuum gas flow in a thruster nozzle, a two dimensional axisymmetric compressible Navier-Stokes (N-S) equations including a continuity, a momentum, an energy, and a turbulence equation are adopted in a vector form as given in Eq (1).

$$\frac{\partial Q}{\partial t}+\frac{\partial (E-E_{V})}{\partial z}+\frac{\partial (F-F_{V})}{\partial r}=0$$ (1)

Here, *r* and *z* are the radial and axial coordinates [2,3]. *E*_*v*_ and *F*_*v*_ are the viscous flux vectors in the *z* and the *r* direction. Also, *Q*, *E* and *F* are the conservation variables of the flowfield and the inviscid flux vectors in Eq (2) [2,3].

$$E_{V}=\left(\begin{array}{c} 0\\ \tau_{zz}\\ \tau_{zr}\\ u\tau_{zz}+v\tau_{zr}-q_{z}\\ \tau_{kz}\\ \tau_{wz} \end{array}\right)\;F_{V}=\left(\begin{array}{c} 0\\ \tau_{zr}\\ \tau_{rr}\\ u\tau_{zr}+v\tau_{rr}-q_{r}\\ \tau_{kr}\\ \tau_{wr} \end{array}\right)$$ (2)

$$Q=\left(\begin{array}{c} \rho \\ \rho u\\ \rho v\\ \rho e\\ \rho k\\ \rho \omega \end{array}\right)\;E=\left(\begin{array}{c} \rho u\\ \rho u^{2}+p\\ \rho uv\\ \rho uh\\ \rho uk\\ \rho u\omega \end{array}\right)\;F=\left(\begin{array}{c} \rho v\\ \rho uv\\ \rho v^{2}+p\\ \rho vh\\ \rho vk\\ \rho v\omega \end{array}\right)$$

The variables *ρ*, *p*, *u*, *v*, *h*, and *e* indicate the density, pressure, axial and radial velocities, enthalpy, and total energy, respectively. Also, turbulent kinetic energy and specific dissipation rate are symbolized as *k* and *ω*, while shear stresses and heat fluxes are denoted as *τ* and *q* [2,3].

Total energy, enthalpy and the pressure of flowfield are given by the following equations [2,3].

$$e=\sum_{i=1}^{N_{i}}Y_{i}h_{i}-\frac{p}{\rho }+\frac{1}{2}(u^{2}+v^{2})=\sum_{i=1}^{N_{i}}Y_{i}\int C_{p,i}(T_{i})dT-\frac{p}{\rho }+\frac{1}{2}(u^{2}+v^{2})$$ (3)

$$p=\rho RT\sum_{i=1}^{N}\frac{Y_{i}}{W_{i}}$$ (4)

Here, *Y*_*i*_ is the mass fraction of the product gases and *R* is the universal gas constant (8314.41 J/kmol K). Also, the shear-stress transport (SST) *k−ω* turbulence model was adopted to consider the turbulence effect near the thruster nozzle wall. For a numerical calculation, the governing equation, Eq (1), was discretized and integrated over all the grid cells based on the finite-volume method, and then solved implicitly [2,3].

### Direct Simulation Monte Carlo method

In general, the continuum flow model such as the Navier-Stokes equation has inherent limitation in application to the rarefied flow regime because an assumption of local thermodynamic equilibrium becomes less valid. In such case, the Boltzmann equation in Eq 5 should be solved statistically to predict the rarefied flow behaviors.

$$\frac{\partial }{\partial t}(nf)+\vec{v}\frac{\partial }{\partial \vec{r}}(nf)+\vec{F}∙\frac{\partial }{\partial \vec{v}}(nf)=\int_{-\infty }^{\infty }\int_{0}^{4\pi }n^{2}(f^{*}f_{1}^{*}-ff_{1})v_{r}\sigma d\Omega d\vec{v_{1}}$$ (5)

Among various statistical methods, the Direct Simulation Monte Carlo (DSMC) method suggested by Prof. Bird [4] is famous for its effectiveness for solving the Boltzmann equation. The DSMC method is a particle simulation technique based on kinetic theory by modeling the behaviors of the number of representative simulated particles statistically and tracing them in the calculation domain during a finite time scale. General procedures of the DSMC method consist of four major steps with moving, indexing, collision and sampling. During each time step, a number of simulated particles travel in the discretized computational domain, and exchange internal energy through a collision with each other or with the given boundary surface. Finally, their velocity components, position coordinates and internal states are calculated and then macroscopic flow properties are evaluated in the form of averages over all simulated particles [4]. Although DSMC is a common method in the investigation of rarefied gas flows, it requires a considerable memory demands because it uses simulated particles to model the movement and the collision of the real gas flows. To overcome this, the development of a unified gas kinetic scheme which can treat continuum-rarefied gas flows together in one method is a challenge in the field of computational fluid dynamics [5,6].

In the present study, a parallel DSMC code was adopted for the present three dimensional unstructured cells to model a complicated geometry of the actual satellite configuration. The variable hard sphere (VHS) model was used to consider intermolecular collisions between the simulated particles, and the no time counter (NTC) method was applied for the collision sampling [4]. Also, the Larsen–Borgnakke phenomenological model was used to calculate energy redistributions energy between translational and internal energy modes [7]. To minimize statistical scatter, macroscopic properties of the plume flowfield were obtained by averaging over sampling steps about 50,000. A steady state is assumed after 5,000 transient steps and 10,000 sampling steps are additionally performed to get the time averaged flow properties.

## Results and discussion

Although the DSMC method can accurately describe the rarefied flow dynamics, some difficulties exist due to the limitations of the cell size and time step, as well as the corresponding high computer memory and speed demands to consider the numerous numbers of representative simulated particles. To consider the numerical efficiencies of the analysis such as the accuracy, calculation time, etc., the present study divided the entire simulation region into three different subdomains based on the flow regimes: a stagnation condition of a combustion chamber, a continuum flowfield in a thruster nozzle, and a rarefied plume flowfield in a vacuum space outside the nozzle, respectively. To analyze these different flow regimes, various numerical methods were applied to each subdomain sequentially by combining a calculated result from one method as the initial or boundary conditions for the other ones. Therefore, the combination of N-S equations and the DSMC method was employed to increase the overall efficiency of the numerical calculations as follows.

### Continuum flow inside nozzle

For a stagnation condition in the thruster chamber, the chemical equilibrium reaction of the fuel and oxidizer was considered to predict the chemical species composition of the propellant combustion gas and the adiabatic flame temperature. A combination of monomethylhydrazine (MMH, *CH*_*3*_*N*_*2*_*H*_*3*_) and nitrogen tetroxide (NTO, *N*_*2*_*O*_*4*_) was chosen in the present study because it is actually preferred for the space applications. A global chemical equilibrium reaction of MMH and NTO is given as Eq (6), where *α* is Stoichiometric coefficient and *n*_*pi*_ is the mole number of each product species [2,8,9].

$$4\alpha {CH}_{3}N_{2}H_{3}+5N_{2}O_{4}\to n_{p1}CO_{2}+n_{p2}H_{2}O+n_{p3}O_{2}+n_{p4}N_{2}+n_{p5}NO+n_{p6}CO+n_{p7}OH+n_{p8}H_{2}+n_{p9}O+n_{p10}H+n_{p11}N+n_{p12}NO_{2}+n_{p13}H_{2}O_{2}+n_{p14}HO_{2}+n_{p15}HNO$$ (6)

The following mass conservation equations of the major elements (*C*, *O*, *H*, and *N*) and the equilibrium constants of eleven elementary equilibrium equations are solved with Eq (6) simultaneously [2,8,9].

$$\begin{array}{l} n_{p1}+n_{p6}-4\alpha =0\\ 2n_{p2}+n_{p7}+2n_{p8}+n_{p10}-24\alpha =0\\ 2n_{p1}+n_{p2}+2n_{p3}+n_{p5}+n_{p6}+n_{p7}+n_{p9}+2n_{p12}+2n_{p13}+2n_{p14}+n_{p15}-20=0\\ 2n_{p4}+n_{p5}+n_{p11}+n_{p12}+n_{p15}-8\alpha -10=0\\ n_{p5}^{2}-K_{p7}n_{p4}n_{p3}=0\\ n_{p6}^{2}n_{p3}P_{r}-K_{p1}n_{p1}^{2}n_{t}=0\\ n_{p7}^{2}n_{p8}P_{r}-K_{p2}n_{p2}^{2}n_{t}=0\\ n_{p8}^{2}n_{p3}P_{r}-K_{p3}n_{p2}^{2}n_{t}=0\\ n_{p9}^{2}P_{r}-K_{p5}n_{p3}n_{t}=0\\ n_{p10}^{2}P_{r}-K_{p4}n_{p8}n_{t}=0\\ n_{p11}^{2}P_{r}-K_{p6}n_{p4}n_{t}=0\\ n_{p12}^{2}n_{t}-K_{p8}n_{p4}n_{p3}^{2}P_{r}=0\\ n_{p13}n_{t}-K_{p9}n_{p8}n_{p3}P_{r}=0\\ n_{p14}^{2}n_{t}-K_{p10}n_{p8}n_{p3}^{2}P_{r}=0\\ n_{p15}^{2}n_{t}-K_{p11}n_{p4}n_{p8}n_{p3}P_{r}=0\\ n_{p1}+n_{p2}+n_{p3}+n_{p4}+n_{p5}+n_{p6}+n_{p7}+n_{p8}+n_{p9}+n_{p10}+n_{p11}+n_{p12}+n_{p13}+n_{p14}+n_{p15}-n_{t}=0 \end{array}$$ (7)

Eqs (8) ~ (10) show total number of product gas moles (*n*_*t*_) and the equilibrium constant (*K*_*pi*_) which is a function of Gibbs free energy (*G*), enthalpy (*h*), and entropy (*S*) under standard condition, respectively [2,8,9].

$$n_{t}=\sum_{i}n_{pi}$$ (8)

$$K_{pi}={exp}^{-(\frac{\Delta G}{RT})}$$ (9)

ΔG=Δh−TΔS (10)

Also, specific heat capacities of gases (*C*_*p*_), enthalpies (*h*), and entropies (*S*) can be determined as polynomial forms of temperature [10].

$$\frac{C_{p}}{R}=a_{1}+a_{2}T+a_{3}T^{2}+a_{4}T^{3}+a_{5}T^{4}$$ (11)

$$\frac{H}{RT}=a_{1}+\frac{a_{2}}{2}T+\frac{a_{3}}{3}T^{2}+\frac{a_{4}}{4}T^{3}+\frac{a^{5}}{5}T^{4}+\frac{a_{6}}{T}$$ (12)

$$\frac{S}{R}=a_{1}\;\ln\;T+a_{2}T+\frac{a_{3}}{2}T^{2}+\frac{a_{4}}{3}T^{3}+\frac{a_{5}}{4}T^{4}+a_{7}$$ (13)

Adiabatic flame temperature and molecular mass (*M*) of the combustion product gas mixture were calculated following Eqs (14) and (15) after mole numbers of all gas species were obtained [2,8,9].

$$\Delta H_{r}^{{}^{\circ}}=\sum_{i}n_{pi,products}\;\int_{298}^{T_{ad}}C_{pi}dT$$ (14)

$$M=\frac{\sum n_{i}M_{i}}{\sum n_{i}}$$ (15)

The final stagnation flow conditions of the bipropellant combustion gas inside the thruster chamber are summarized as Table 1.

**Table 1:** Chemical equilibrium reaction result of MMH-NTO propellant.

Results	MMH-NTO
Mole fractions of combustion gas species	
*H*_*2*_*O*	0.3242
*N*_*2*_	0.3041
*H*_*2*_	0.1561
*CO*	0.1311
*CO*_*2*_	0.0362
Other species	< 0.01
Molecular mass of gas mixture [g/mol]	20.40
Adiabatic flame temperature [K]	3056

**Fig 2:**
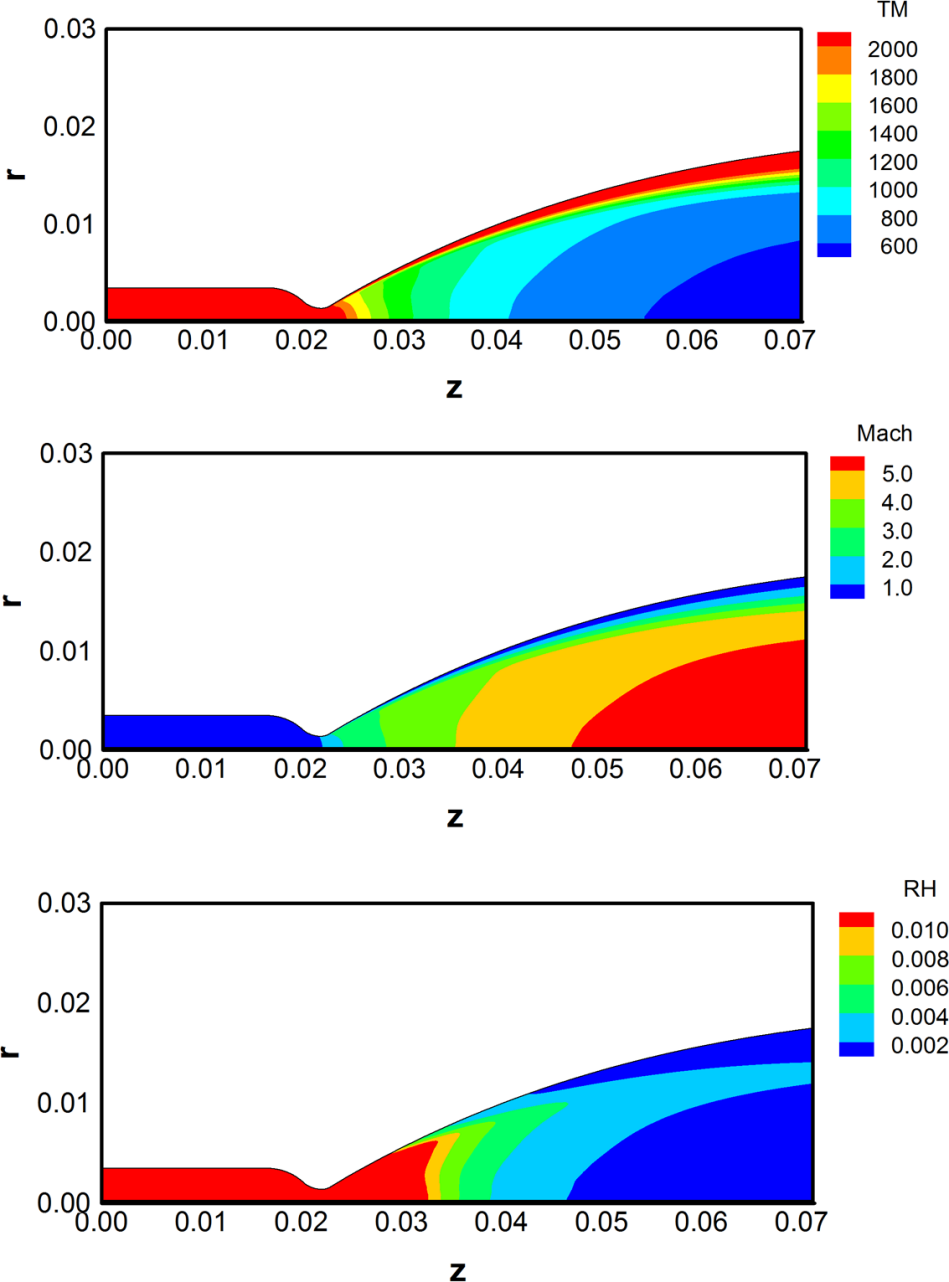
Continuum flow results inside the thruster using N-S equations. (A) Temperature [K]. (B) Mach number. (C) Density [kg/m^3^].

**Fig 3:**
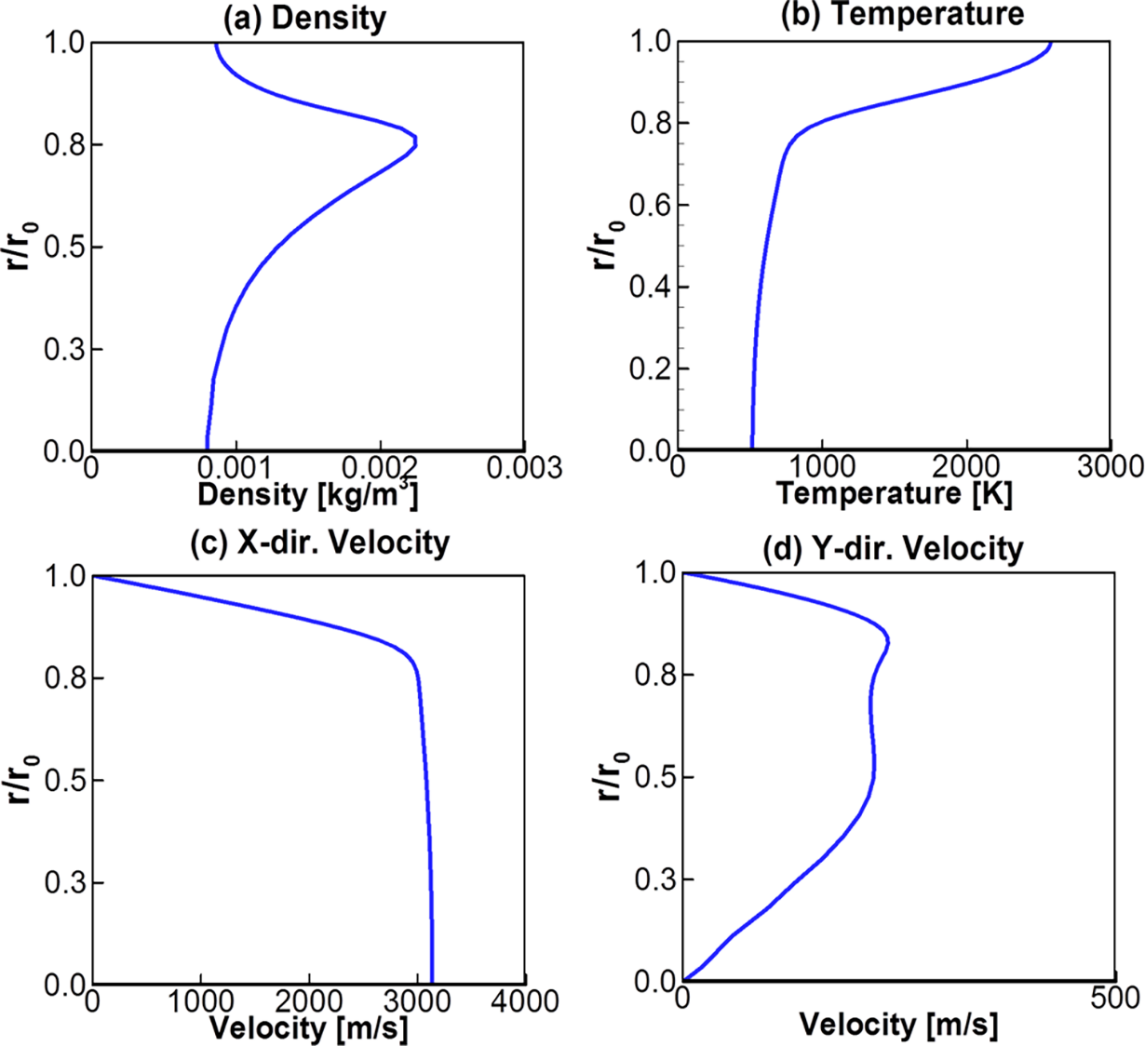
Exhaust plume flow properties at the thruster nozzle exit plane.

For the inlet condition of the DSMC method, the computational fluid dynamics (CFD) method based on the N–S equations in Eq (1) was solved to obtain the plume flowfields at the thruster nozzle exit plane. The calculation grid was generated with a total 150×41 = 6,150 nodes along the nozzle axis and radius inside the thruster. For the nozzle inlet condition and the initial chamber condition of the N-S equations, the stagnation flow data obtained by the chemical equilibrium reaction were applied shown in Table 1. The product gas species were assumed to be a mixture of ideal gases, and their compositions were fixed (chemically frozen) through a nozzle expansion. In addition, adiabatic and no-slip conditions were applied on the nozzle wall, and an extrapolation was imposed at the nozzle exit plane for an outflow boundary condition from the flow properties of the interior domain. Fig 2 shows the major calculated results of the continuum nozzle flow inside the bipropellant thruster. It could be predicted that the temperature of the expanded gas flow in Fig 2A was over 2,400 K near the nozzle wall and 500 K at the center region of the nozzle exit, respectively, during a supersonic expansion process as the adiabatic stagnation temperature in the chamber exceeded 3,000 K shown in Table 1. Finally, it was accelerated to the supersonic gas flow above Mach number 5 as it moved closer to the nozzle exit plane seen in Fig 2B. In the case of the density distribution of the gas flow, it especially tended to be decreased near the nozzle wall and at the center region of the nozzle exit shown in Fig 2C. The final bipropellant gas flowfields at the nozzle exit plane are illustrated in Fig 3 including the density, temperature, axial and radial velocity components. Some dramatic variations in the flow properties are found when approaching the nozzle wall because of the viscous boundary layer effect.

### Plume gas flow influence in vacuum space environment

**Fig 4:**
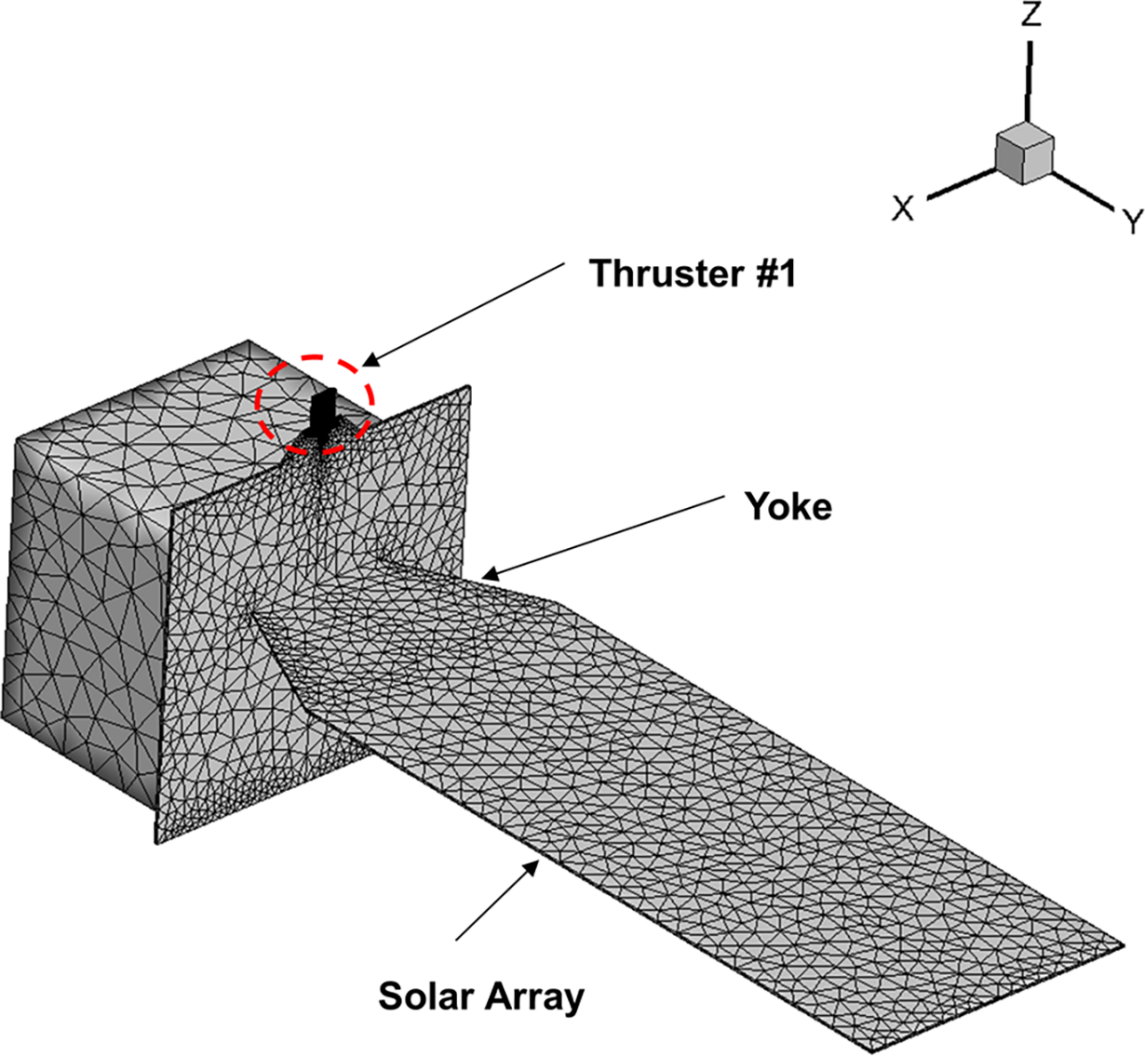
Calculation grid of GEO satellite.

A calculation grid of the GEO satellite considered in this study is illustrated in Fig 4. It features a box-shaped structure and a large thin solar array is connected horizontally to the middle of the satellite body through a yoke. This satellite has a total of seven bipropellant thrusters with 10 N grade. They use the storable propellants consisting of monomethylhydrazine as a fuel and nitrogen tetroxide as an oxidizer. Among seven thrusters, the present study considered the plume flow exhausted from thruster #1 only because a considerable amount of its plume would be impinged directly on the entire area of the solar array as it is located above the center of the solar array’s side edge (*–Y*). The nozzle exit plane of the thruster is not canted outward to the vacuum space so to consider the maximum influences of the plume. To model a vacuum boundary condition, the size of the computational domain is set to 6 m in width (*x*) and height (*z*) and 10.0 m in length (*y*) for each. The computational grid is developed using the unstructured tetrahedral cells following the one-third rule, which means all the cell sizes were adjusted less than at least one third of the local mean free based on the Knudsen number prediction of the plume flow. To find an optimum cell number, a grid sensitivity study was conducted simply to check accuracy of the present results. A coarse, a medium and a fine grid were created using about 120,000, 240,000 and 480,000 cells, respectively. In addition, at least 20 simulated particles per cell were used in each case. The density and overall temperature results of the plume were measured following the axial direction from a center of the nozzle exit and then they were compared. In case of the plume density, these results show almost same profiles regardless of the number of cells. On the other hand, some deviations can be found for the overall temperature results depending on the cell number. The average and maximum relative error between the overall temperature results of the coarse and the fine grids are estimated within 10% and 15% deviations, while those errors are found to have smaller deviations within 3% and 5% between the medium and the fine grids, respectively. From this, it can be judged that the deviations become smaller as the number of cells increases, and thus a certain level of consistency in the accuracy can be observed. Also, it is predicted from this grid sensitivity study that the accuracy would not be improved drastically although the cell numbers increase further in the order of the hundreds of thousands. The accuracy would be improved slightly with increased cell numbers but a great amount of computing time will be also required additionally. Thus, after considering the computing time and the accuracy together, it was decided that it would be proper that the final estimations of the plume influences on the solar array of the GEO satellite are analyzed using about 480,000 cells, which can be taken as the best optimized result among all of the calculations.

For the DSMC simulation in the present study, the nozzle exit of the given thruster was assumed as an interface plane between the continuum and the rarefied flow domains, which is a reasonable assumption for most rocket nozzles [11]. From the general viewpoint, the Chapman-Enskog distribution function is a natural choice for modeling particles coming into the DSMC computational domain at the interface boundary between the Navier-Stokes and DSMC domains since it is consistent with the Navier–Stokes equations and provides the theoretically accurate results. However, some researchers have chosen to apply a Maxwellian distribution in hybrid Navier Stokes-DSMC simulations since the use of a Chapman–Enskog distribution leads to a small improvement in accuracy compared to the Maxwellian distribution [12–15] while its use would greatly increase the computational cost since more random number calls and computational operations are required [16], or because of the fact that the Chapman–Enskog distribution becomes negative with deviation from the Maxwellian distribution [12,13], or because of the observation that, for specific flows, a greater mismatch at the interface between Navier-Stokes and DSMC was observed for a Chapman–Enskog distribution as compared to a Maxwellian distribution [17]. Also, the comparison with the solution of the Burnett equation didn't reveal any problem with the usage of the Maxwellian distribution function when the Maxwellian distribution function in conjunction with kinetic-moment boundary conditions was used in the DSMC method [18]. Moreover, for a high-speed gaseous jet or plume flow from a planar or annular exit, it is considered as common and reasonable to assume that gas at the exit is in an equilibrium state [19]. This assumption of the equilibrium flow conditions for the DSMC inflow has introduced no considerable distortion into the plume near field [20]. Thus, several researchers have employed the simple approach to Navier-Stokes and DSMC coupling based on the Maxwellian distribution and shown that it worked quite well for the nozzle and plume studies in their works [20–28]. As the present study employed a simple one-way coupling at the interface between the Navier-Stokes and DSMC domains, the present study assumed that the gas is at equilibrium state at the nozzle exit based on the reference [19]. As a consequence, the plume particles at the inflow interface of the DSMC have been generated according to a Maxwellian distribution function based on the flow parameters from the Navier–Stokes solution to simplify the problem although the Chapman-Enskog distribution function would be the most accurate. Thus, the continuum flow properties at the nozzle exit by the N-S equations in Fig 3 were applied directly as the inflow boundary condition for the present DSMC method together with the chemically frozen gas mixture compositions given in Table 1 using the Maxwellian distribution function. In addition, a single weight is assumed for all the gas species during the DSMC calculation. The representative gas properties used for the VHS model are summarized in Table 2 [4].

**Table 2:** Gas properties for VHS model (Reference temperature: 273 K) [4].

Speices	Diameter (*d*☓10^10^ m)	Mass (*m*☓10^27^ kg)	Viscosity index	Rotational degree of freedom
*H*_*2*_*O*	4.51	29.9	0.75	3
*N*_*2*_	4.17	46.5	0.74	2
*H*_*2*_	2.92	3.34	0.67	2
*CO*	4.19	46.5	0.73	2
*CO*_*2*_	5.62	73.1	0.93	2

**Fig 5:**
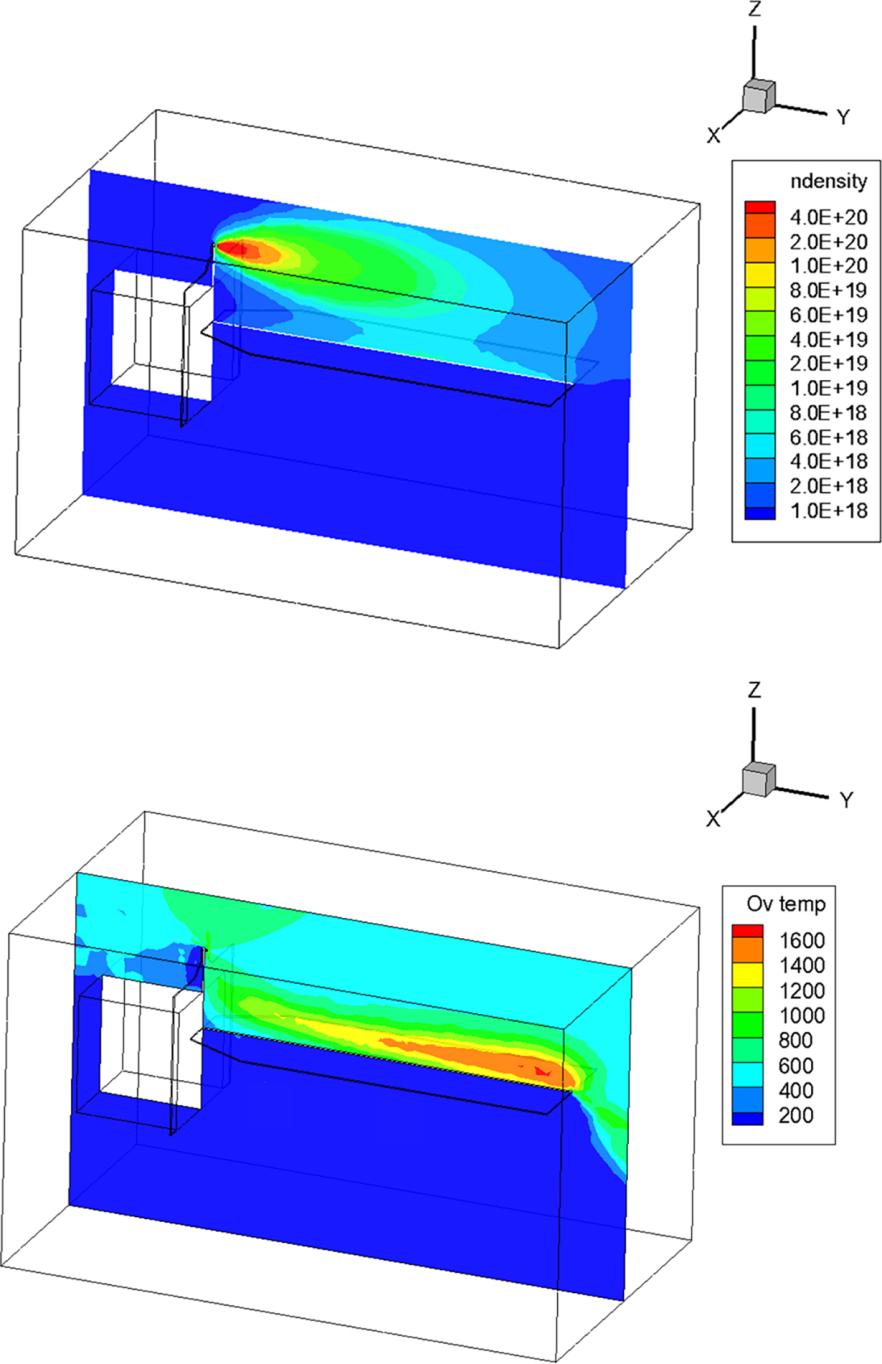
DSMC analysis results of overall plume gas flow behaviors. (A) Number density [m^-3^]. (B) Overall Temperature [K].

As the first result, the number density and overall temperature of the plume flow were investigated. The number density distribution of the plume flow at the center cross section of the calculation domain is illustrated in Fig 5A. The simulated plume particles were initially ejected from the thruster #1 nozzle exit, and also, a considerable amount of the plume gas directly collided on the upper side of the solar array and then reflected from it. Thus, the number density distribution of the plume flow was increased over an order of 6.0E+18 m^-3^ near the solar array surface whereas it was decreased to less than 1.0E+18 m^-3^ in the empty space. This high number density distribution also yielded an increase of the overall temperature of the plume gas seen in Fig 5B. A higher gas temperature region was found around the upper region of the solar array far from the satellite. However, the temperature in the space decreased gradually because of the rapid expansion of the plume gas flow in the vacuum condition. The temperature increased from 600 K at the core of the plume flow to 1,600 K around the solar array because a considerable amount of the kinetic energy of the plume gas particles was converted into thermal energy during intensive collisions with each other. Thus, adverse influences including a disturbance force/torque, heat load, and chemical species deposition can be predicted at the upper surface of the solar array due to a direct plume impingement.

**Fig 6:**
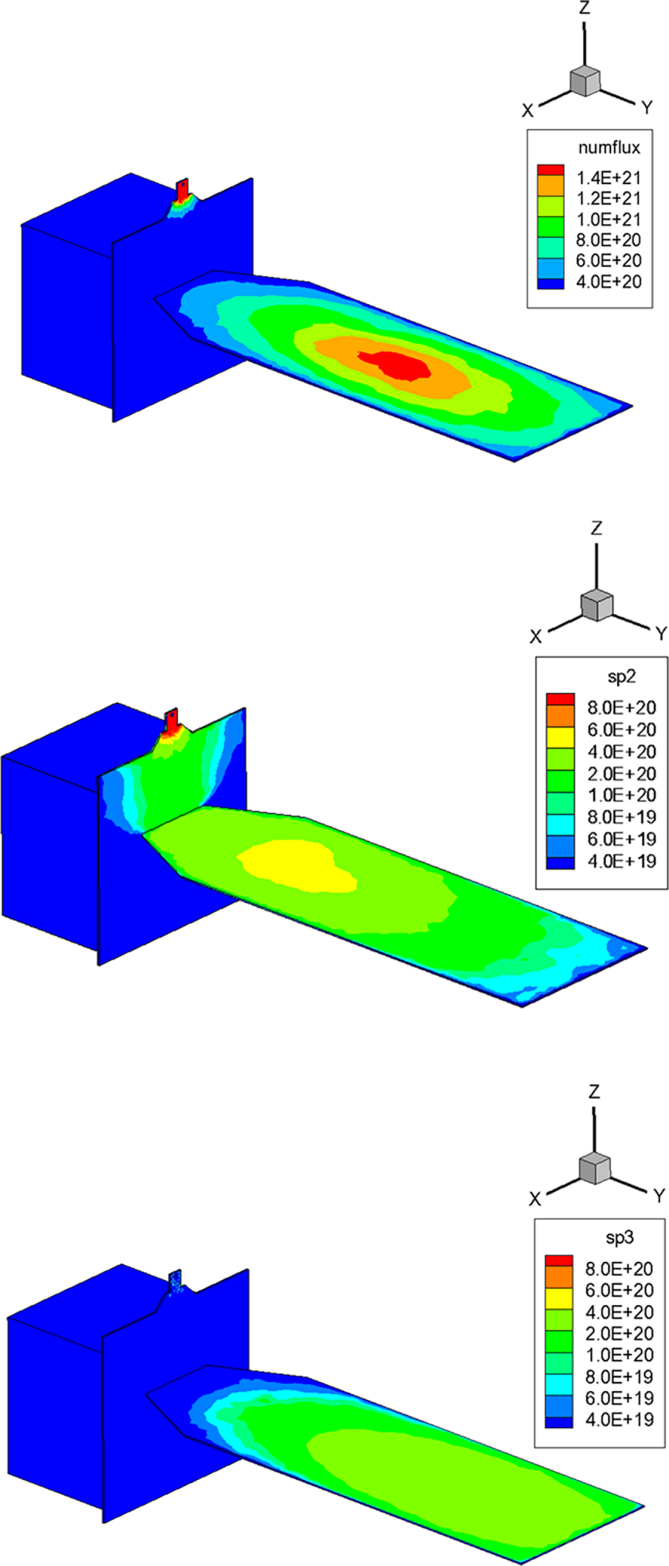
Surface distribution of exhaust plume gas flow [m^-2^·s]. (A) Overall gas mixture. (B) *H*_*2*_ species. (C) *N*_*2*_ species.

Next, the surface distributions of the plume gas mixtures were predicted shown in Fig 6. It was observed from Fig 6A that a large portion of the overall plume gas, which was ejected from thruster #1, collided on the upper surface of the solar array because thruster #1 was located above the center of the solar array’s side edge (*–Y*). The order of the maximum number flux was estimated over 1.4E+21 m^-2^·s at the center region of the solar array. Individual number fluxes of the major species, *H*_*2*_ and *N*_*2*_, are summarized in Fig 6B and 6C, which show similar tendencies between their overall distributions over the entire area of the solar array. However, it was found that the maximum value of the number flux decreased inversely proportional to the molecular mass of the species, which predicted roughly 4E+20 m^-2^·s for *H*_*2*_ and 2E+20 m^-2^·s for *N*_*2*_. Especially in the case of *H*_*2*_, it also spread more widely on the vertical panel of the satellite where the solar array is connected. A possible reason is that the *H*_*2*_ could be separated easily from the main stream of the plume flow during the rapid expansion process due to its lighter molecular mass.

**Fig 7:**
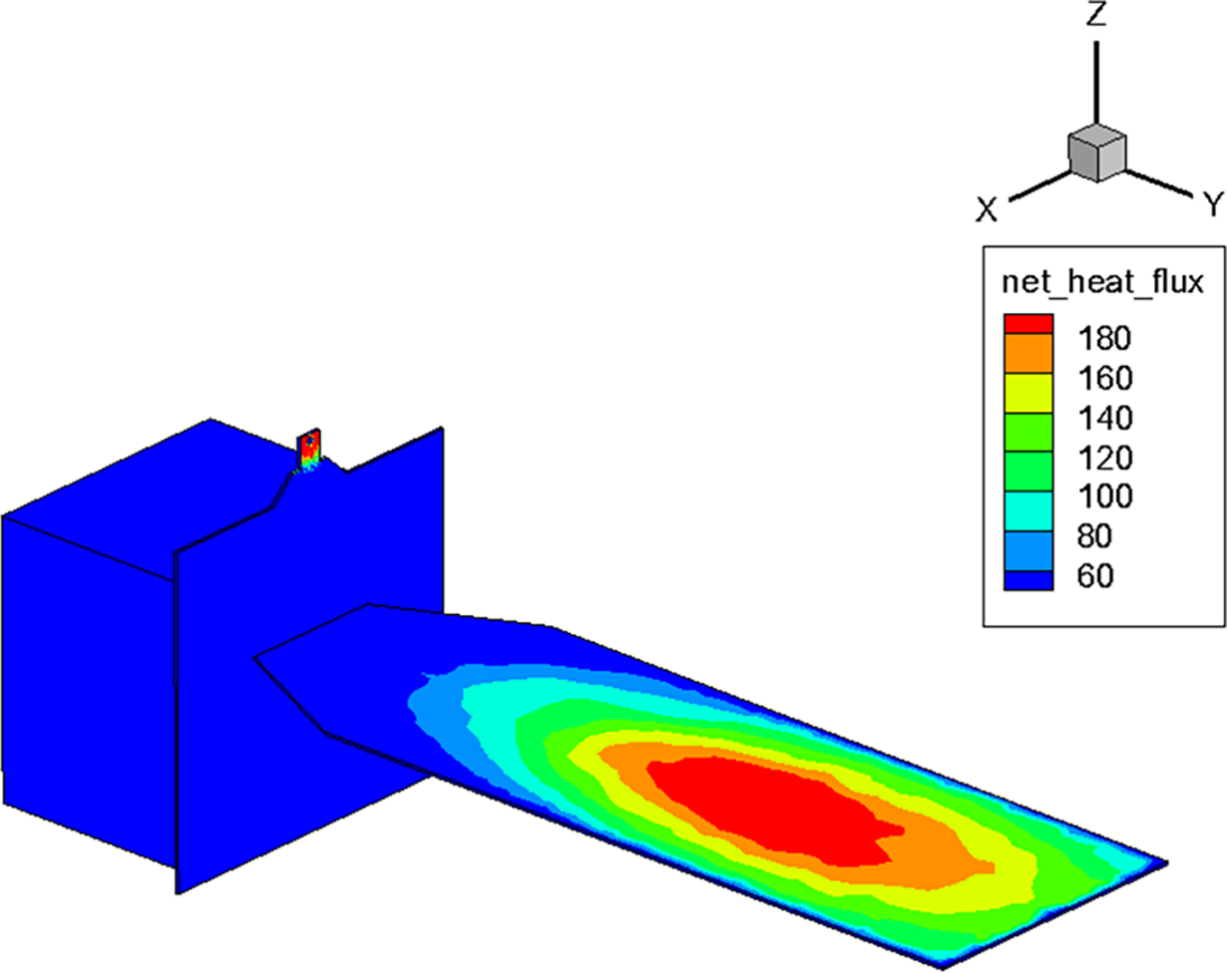
Surface heat flux distribution of exhaust plume gas flow [W/m^2^].

To predict the heating effect on the satellite surfaces by the collision of the plume gas particles, the coldest temperature of the solar array at 323 K, which was estimated from a thermal analysis of the satellite in a mission orbit, was used as a wall boundary temperature for the present DSMC simulation. The surface heat flux estimation by the plume on the solar array is illustrated in Fig 7, which showed a similar distribution with the number flux result of the plume species in Fig 6A because more collisions of the plume gas particles can deliver a larger thermal energy on the surface of the solar array. The maximum heat flux on the solar panel was predicted to be about 220 W/m^2^, however, it is relatively low when compared with the solar constant value *q*_sol_ of 1353 W/m^2^ [29,30]. In addition, the contamination influence of the plume gas species on the solar array was evaluated to be negligible because the surface temperature of the solar array was much higher than the capturing temperatures of the plume gas particles on the satellite surface.

Moreover, the estimated disturbance force and torque values caused by the thruster plume are summarized in Table 3. It could be predicted that a considerable portion of the disturbance force and torque would occur due to a direct collision of the plume particles on the solar array. From Table 3, the disturbance force on the *Y*-axis and the disturbance torque on the -*X*-axis were particularly large for the present configuration of the GEO satellite when thruster #1 was fired.

**Table 3:** Predictions of disturbance force and torque values on the solar array.

Disturbance Force [N]	X	Y	Z
	2.61E-04	8.28E-01	-4.83E-01
Disturbance Torque [N· m]	X	Y	Z
	-2.58E+00	3.76E-03	4.93E-03

**Fig 8:**
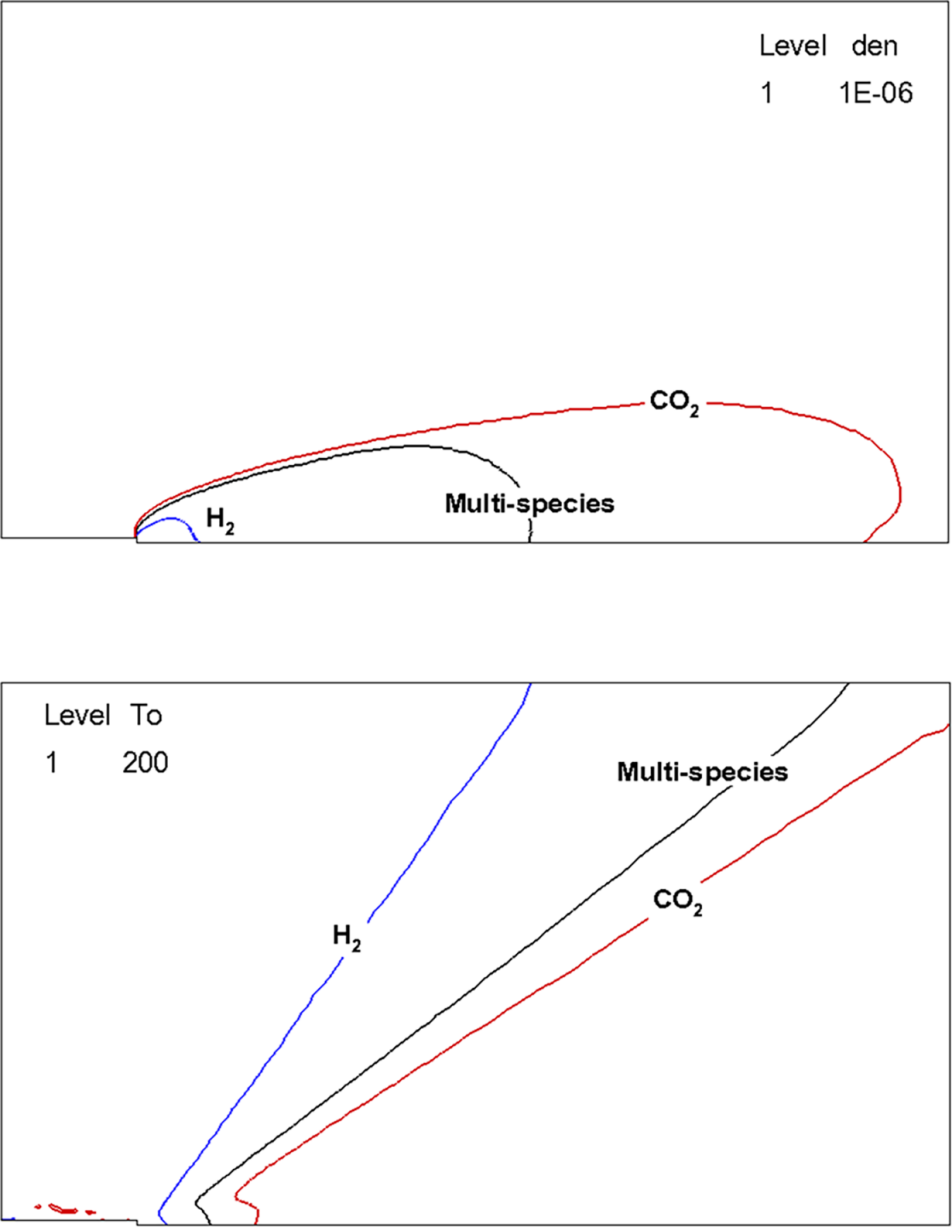
Plume behavior comparison of single and multi-species gas in the main stream zone. (A) Density [kg/m^3^]. (B) Overall Temperature [K].

**Fig 9:**
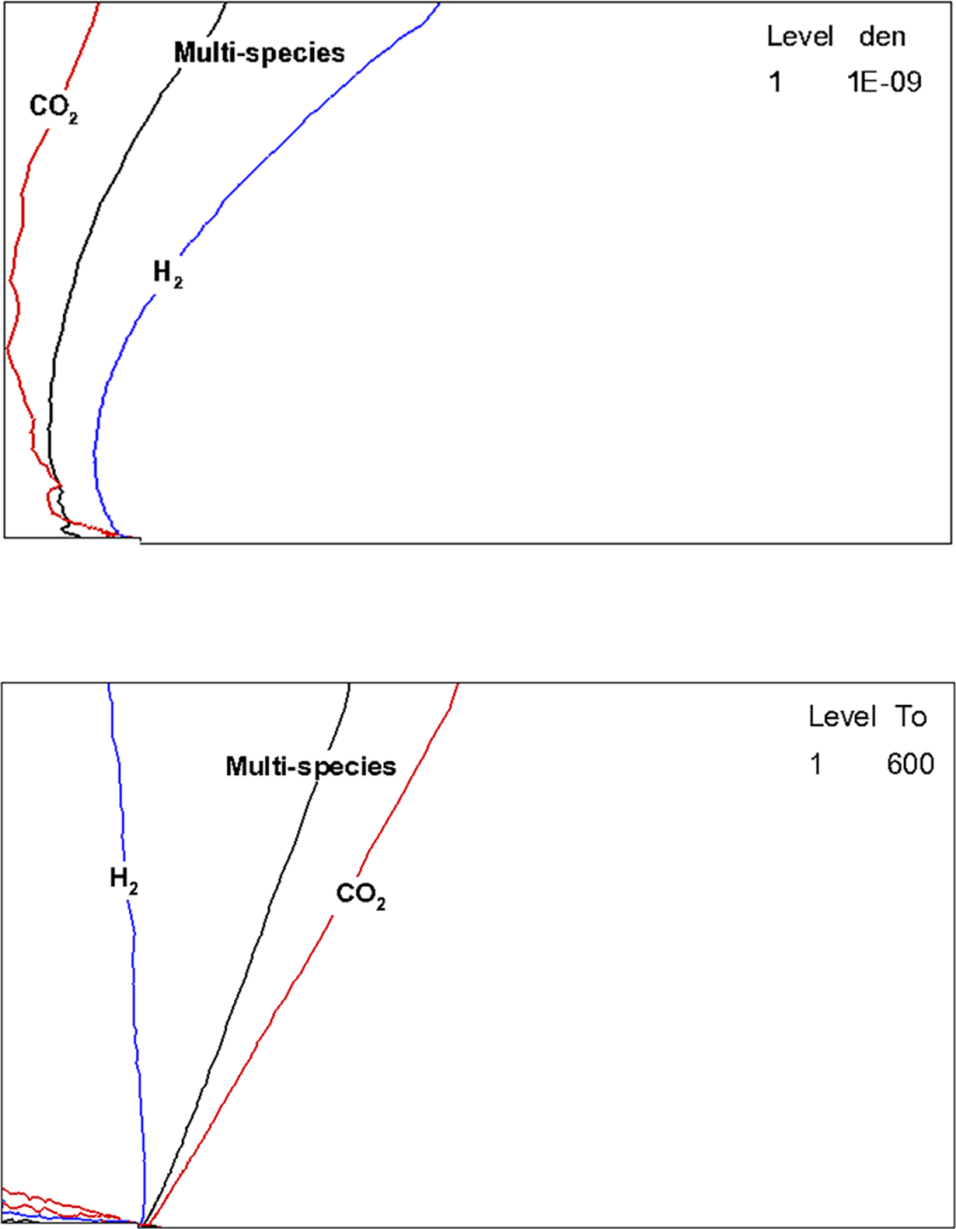
Plume behavior comparison of single and multi-species gas in the backflow stream zone. (A) Density [kg/m^3^]. (B) Overall Temperature [K].

As a final result, the effects of bipropellant multi-species gas are discussed. When assuming the exact same thermodynamic nozzle exit conditions with a single gas species, the major differences of the plume flow behaviors can depend greatly on the molecular weight of the gas species. To observe clearly, additional calculations were conducted in the 2-D axisymmetric domain with a single *H*_*2*_ and *CO*_*2*_ gas species, respectively. And then, they were compared with the present bipropellant multi-species gas mixture because *H*_*2*_ represents the lightest species while *CO*_*2*_ is the heaviest one among the bipropellant plume gas species in Table 1. In the case of the main plume stream zone, it is seen from Fig 8A that the heavier gas species tended to diffuse more widely due to its higher mass momentum effect of the supersonic exhaust plume flow while the lightest *H*_*2*_ species was concentrated on the region adjacent to the nozzle exit. As a result, the overall temperature profiles in Fig 8B also showed a larger discrepancy with the present multi-species gas temperature result. Therefore, the plume impinging effects on the present solar array configuration, such as the heat flux and the disturbances, could be more severe for the heavier gas species than the light one. Otherwise, it could be found from Fig 9A and 9B that a denser and hotter distribution of *H*_*2*_ was observed in the backflow region among the other gases because of its light molecular weight. Thus, the plume impinging effects on the satellite payloads in the present configuration would be more dominant than that of the other heavier gas species.

## Conclusions

In the present study, a numerical analysis of the plume influence of a small MMH-NTO bipropellant thruster was performed for the solar array of a GEO satellite. To deal with the complex plume flow regimes of the stagnation, continuum, and rarefied conditions efficiently, the combined approach of computational fluid dynamics (CFD) and Direct Simulation Monte Carlo (DSMC) was used depending on the flow conditions. The numerical analysis results show that a considerable amount of the plume particles directly collided on the upper side of the solar array surface and reflected from it because thruster #1 was located above the center of the solar array’s side edge (*–Y*). Thus, the number density distribution and overall temperature of the plume flow increased near the solar array surface. Moreover, the surface distributions of the plume gas mixtures and heat flux by them also affected the entire area of the solar array. As a result, adverse influences including the disturbance force/torque, heat load, and chemical species deposition could be predicted distinctly at the upper surface of the solar array due to a direct plume impingement. In addition, for the multi-species gas mixture, the overall plume effects are caused by the combination of the various gas species behaviors, and also, their individual influence can be evaluated separately while the single gas species is solely responsible for all the plume effects. Thus, the simple assumption of a single gas species can provide unrealistic predictions, which may lead to a wrong design philosophy during satellite development.

Consequently, the results of the present investigation on the plume influences are expected to provide quantitatively useful information for the review and evaluation of the design validity of the given GEO satellite. In the future, a further investigation of the thruster plume flowfields depending on the detailed multi-step chemical reactions between MMH and NTO will be introduced to predict more realistic behaviors of the plume gas species.
